# Surface fractal dimension, water adsorption efficiency, and cloud nucleation activity of insoluble aerosol

**DOI:** 10.1038/srep25504

**Published:** 2016-05-03

**Authors:** Ari Laaksonen, Jussi Malila, Athanasios Nenes, Hui-Ming Hung, Jen-Ping Chen

**Affiliations:** 1 Finnish Meteorological Institute, 00101 Helsinki, Finland; 2 Department of Applied Physics, University of Eastern Finland, 70211 Kuopio, Finland; 3 School of Earth and Atmospheric Sciences, Georgia Institute of Technology, Atlanta, GA 30332, USA; 4 School of Chemical & Biomolecular Engineering, Georgia Institute of Technology, Atlanta, GA, 30332, USA; 5 Institute for Environmental Research & Sustainable Development, National Observatory of Athens (NOA), I. Metaxa & Vas. Pavlou, 15236 Palea Penteli, Greece; 6 Institute of Chemical Engineering Sciences, Foundation for Research and Technology Hellas, Stadiou Str., Platani, GR-26504 Patras, Greece; 7 Department of Atmospheric Sciences, National Taiwan University, Taipei 10617, Taiwan

## Abstract

Surface porosity affects the ability of a substance to adsorb gases. The surface fractal dimension *D* is a measure that indicates the amount that a surface fills a space, and can thereby be used to characterize the surface porosity. Here we propose a new method for determining *D*, based on measuring both the water vapour adsorption isotherm of a given substance, and its ability to act as a cloud condensation nucleus when introduced to humidified air in aerosol form. We show that our method agrees well with previous methods based on measurement of nitrogen adsorption. Besides proving the usefulness of the new method for general surface characterization of materials, our results show that the surface fractal dimension is an important determinant in cloud drop formation on water insoluble particles. We suggest that a closure can be obtained between experimental critical supersaturation for cloud drop activation and that calculated based on water adsorption data, if the latter is corrected using the surface fractal dimension of the insoluble cloud nucleus.

Cloud droplets are formed when supersaturated water vapour starts condensing on aerosol particles, or cloud condensation nuclei (CCN). The threshold (or critical) supersaturation at which the activation of CCN to cloud droplets takes place depends on the CCN material. Water soluble aerosols are in general more efficient CCN than insoluble aerosols, and exhibit lower critical supersaturations. However, at low temperatures many insoluble particle types such as mineral dusts are efficient nuclei for ice clouds. In the so called deposition nucleation, ice is formed by direct condensation of water vapour on the aerosol without an intermediate liquid phase. Current theories are unable to predict the critical supersaturations required for deposition nucleation to take place^1^. It is quite obvious that elucidation of ice deposition nucleation onto mineral dusts is difficult if even the liquid drop activation by the dust particles is not well understood. In this paper, our aim is to provide an improved description of the liquid drop activation by incorporating the effect of mineral dust porosity into the CCN activation theory.

The so called Frenkel–Halsey–Hill (FHH)-adsorption–activation theory^2^ has been applied by several authors in recent years to calculate critical supersaturations of different types of water-insoluble particles^3^,^4^,^5^,^6^. The theory combines the FHH adsorption isotherm^7^,^8^,^9^ with the Kelvin equation. The FHH isotherm can be written as ln(*S*) = −*AN*^−*B*^, where *S* is saturation ratio, *N* is the surface coverage (or number of monolayers of adsorbate on the adsorbent surface), and *A* and *B* are adsorption parameters describing the strength of molecular interactions between the surface and the adsorbed molecules. With pure van der Waals fluids, *B* = 3, but with most real-world systems the molecular interactions are more complex, and the values of *A* and *B* can in practice only be determined experimentally.

When combined with the Kelvin equation, the FHH isotherm reads^2^ ln(*S*) = −*AN*^−*B*^ + 2γ*v*/(*k*_B_*TR*), where γ and *v* denote surface tension and molecular volume of water, respectively, *k*_B_ is the Boltzmann constant, *T* is temperature, and *R* is the radius of the droplet encompassing the insoluble particle. This equation can be used to produce a curve of *S* vs. *R* (or *N*) that has a maximum, marking the critical supersaturation at which the cloud drop is formed. Kumar *et al.*^4^ determined the FHH parameters of different particle types based on measured critical supersaturations, whereas Hatch *et al.*^5^ and Hung *et al.*^6^ determined *A* and *B* based on adsorption measurements, and used the FHH-adsorption–activation theory to predict the critical supersaturations. These two approaches do not produce satisfactory closure. Motivated by this discrepancy, we show how surface structure, expressed by the fractal dimension, and its interaction with water vapour induce the observed critical supersaturations. This allows for an unprecedented degree of understanding of how surface porosity and the associated capillary condensation affect hygroscopic growth and cloud drop activation of water insoluble aerosols in the atmosphere.

## Results

### Fractal dimension and the FHH activation theory

Microscale surface roughness, caused by structures such as pores, slits, kinks, edges, etc., is often described in terms of fractality^10^. A surface can be considered fractal if the structural patterns remain the same regardless of the length scale, i.e. they are self-similar. In such a case, the surface area of an object within a radius *r* is proportional to *r*^*D*^, where *D* is the surface fractal dimension. The value of *D* is always between 2 and 3, the former indicating a perfectly smooth surface, and the latter a surface that fills the space completely. Because gas molecules are suitable “yardsticks” to probe the nano- to microscale surface structures, a number of theories have been proposed for determining *D* based on experimental adsorption isotherms^11^.

The FHH adsorption theory is based on the idea of a potential field caused by the adsorbent surface that acts on the adsorbate molecules. The parameter *A* describes the strength of interaction between the surface and the molecules in the first adsorption layer, and the parameter *B* represents how the interaction decays with distance from the surface. Therefore, the FHH isotherm can always be expressed as ln(*S*) = −*A*(*δ*/*δ*_*m*_)^−*B*^, regardless of the curvature of the surface. Here *δ* is the distance between the surface of the adsorbent, and the surface of the adsorbed layer, and *δ*_*m*_ denotes monolayer thickness. Furthermore, only when the surface is not curved can the term *δ*/*δ*_*m*_ be replaced by *V*/*V*_*m*_, where *V* and *V*_*m*_ denote volumes of the adsorbed layer and the monolayer, respectively. This was already implied by Halsey^8^, who stated that *N* = *V*/*V*_*m*_ can be used in the FHH equation when “the volume adsorbed is proportional to the depth of the layer”. However, even with macroscopically flat surfaces, this proportionality may not hold if they have enough of microstructure so that their fractal dimension *D* is sufficiently different from 2. As adsorption experiments usually measure adsorbed volume rather than adsorption layer thickness, the fractality of the surface may cause the calculated critical supersaturation to be erroneous unless the fractal dimension is correctly accounted for. From here on, we use the notations *N*_*δ*_ = *δ*/*δ*_*m*_ and *N*_*V*_ = *V*/*V*_*m*_.

The proportionality between the volume of a multilayer molecular film on a fractal surface and the film thickness is *V* ~ *δ*^*3-D*^ (see e.g. Avnir and Jaroniec^12^). Thus, the surface coverage in the FHH equation can be written as *N*_*δ*_ = *kN*_*V*_^1/(3-D)^, where *k* is a proportionality constant, and the FHH equation becomes ln(*S*) = −*Ak*^*−B*^*N*_*V*_^−*B/(3-D)*^. This implies that if one wants to determine the FHH parameters based on fitting the FHH equation to an experimental adsorption isotherm (where the adsorbed volume has been measured), the resulting coefficients are in fact *Ak*^*−B*^ and *B/(3-D)* instead of *A* and *B*. However, as the value of *k* has to be unity in the case of a smooth surface with *D* = 2, we assume that *k* = 1 in all cases.

On the other hand, if one is determining the FHH parameters based on fitting the FHH-adsorption-activation-theory to measured critical supersaturations, the resulting coefficients are *A* and *B*. This is because the surface coverage is in this case directly related to *R*, which is a sum of the dry CCN radius and the thickness of the adsorbed film.

None of the dusts considered here is especially hydrophobic, and therefore the adsorbed water is expected to form a multilayer film at the critical supersaturation (in contrast, when the contact angle of water is large, the water layer may consist of tiny droplets^13^,^14^). When fitting the FHH equation to adsorption data in order to obtain the coefficient *B*/(*3*-*D*), one should use a subset of the data that is also clearly in the multilayer regime.

Based on the above consideration, it should be possible to determine the fractal dimension of the surface of an insoluble CCN if both adsorption and critical supersaturation measurements are available. In order to examine the feasibility of this idea numerically, we compare our approach to two traditional methods for determination of *D* (see Methods).

### Experimental determination of surface fractal dimension

The surface fractal dimension was calculated in three different ways for six different mineral particle types. In the first method, we made use of a combination of water adsorption isotherm and CCN activation measurements as explained above. With the thermodynamic and fractal FHH methods, we made use of nitrogen isotherms measured at 77 K (see SI for details). The results are shown in Table 1.

Consider first the two traditional methods for determining *D*. With sodium montmorillonite, calcite, quartz, and Mt. St. Helens (MSH) volcanic dust the agreement is rather good. However, with illite and El Chichon (EC) volcanic dust, the *D*-values differ clearly. A probable reason for this is that there is no unique fractal dimension that would characterize the surfaces of these particular substances properly; i.e. the pore structure is not self-similar at all pore sizes, and the thermodynamic and fractal FHH methods probe somewhat different pore size ranges.

The new adsorption-CCN method produces surface fractal dimensions that are in rather good agreement with the two other methods. Note that a single mineral type exhibits some variability depending on its source that can be caused by complex and to some degree varying chemical composition (as is the case with clay minerals) or surface characteristics (e.g. quartz may have varying surface densities of hydroxyl groups). Thus, if the water and nitrogen adsorption (or CCN) measurements have been carried out with dusts obtained from different sources, a perfect match between the *D*-values cannot be taken as granted. Here, we can be certain only about the similarity of the volcanic dusts in each three types of measurements. In addition, the nitrogen and water adsorption measurements for sodium montmorillonite^6^ and for quartz^16^ were conducted using one batch of adsorbent in both cases.

### Cloud drop activation

**Figure 1:**
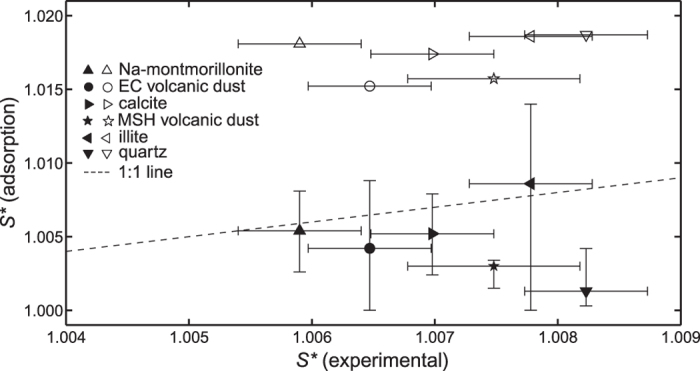
Comparison between experimental critical supersaturations and those calculated using FHH parameters obtained from adsorption measurements, either without (empty symbols) or with (full symbols) fractal correction.

The results shown in Table 1 indicate that the *D*-values determined using the thermodynamic and/or the fractal FHH methods can be used, together with the water adsorption isotherm, to calculate the FHH-parameter *B*, which provides a good approximation of *B*_*CCN*_. Figure 1 shows a comparison between experimental critical supersaturations^4^,^15^, and supersaturations calculated using the *B*-parameters obtained from adsorption measurements (the *A*-values were not changed). The empty symbols are calculated without any corrections to the *B*-values obtained from water adsorption isotherms (i.e. assuming *D* = 2), and the full symbols are calculated with a fractal correction (i.e. assuming that *D* equals the average of *D*_N2_(TD) and *D*_N2_(f-FHH) in Table 1). In all six cases, the agreement between the experimental and the calculated critical supersaturations is better when the fractal correction is made. It is quite probable that much of the remaining disagreement is due to the differences of the mineral structures used in the CCN, water adsorption and nitrogen adsorption experiments. Thus, although the agreement is not perfect in all cases, our results strongly suggest that closure between experimental critical supersaturations and adsorption data can be reached when the surface fractal properties of the aerosols are properly accounted for.

## Discussion

In past studies, fractal dimension of atmospheric aerosol particles has usually referred to the mass fractal dimension, given by the exponent in the relation between number of primary aggregates and the radius of gyration (or mobility size). However, it has been noted^17^ that when characterizing agglomerate particle properties, two fractal dimensions, one describing the overall structure of the agglomerate and another describing the surface structure, are needed. As we have shown, the surface fractal dimension is an important quantity in determining the critical supersaturation of cloud droplets forming on insoluble nuclei. Moreover, it is likely that the same applies to heterogeneous ice nucleation as well. It has been found that deposition ice nucleation and immersion freezing scale with seed particle surface area^18^,^19^,^20^, and accounting for the surface fractal geometry will allow more precise surface area estimation. Recent theoretical works suggest that deposition nucleation is caused by freezing of capillary condensed water in surface pores^21^, or by activation of ice clusters adsorbed on the surfaces of insoluble nuclei^22^. In either case, a measure such as the surface fractal dimension should be useful in the derivation of a quantifiable model. Furthermore, the surface fractal dimension offers a way to quantify immersion mode freezing, as a liquid layer is required to exist first before the immersion nuclei (IN) can manifest their ice nucleation activity. The variability of experiments on the IN freezing efficiency and temperature of freezing may, in part, be due to an incomplete understanding of the formation of a liquid phase on the IN. The theory presented here offers the ability to address that. Apart from cloud formation itself, the interaction between water vapour and porous surfaces is very important for other atmospheric phenomena, such as the dispersion of volcanic ash plumes during eruptions as the activation of ash into cloud droplets or ice crystals can notably increase plume temperature and buoyancy through latent heat release^23^.

## Methods

### Thermodynamic theory of fractal dimension

Neimark^24^ derived a thermodynamic theory of fractal dimension, aimed at quantifying the surface fractal dimension based on adsorption measurements on porous surfaces. This theory has the advantage that it does not rely on any particular adsorption isotherm. It is based on the concept of capillary condensation: as the saturation ratio increases, progressively larger pores are filled by the adsorbate, as dictated by the Kelvin equation*: a*_*c*_ = *−2γv/(k*_B_*T* ln *S*), where *a*_*c*_ is a characteristic radius of the pores filled at saturation ratio *S*. The total surface area covered at the same saturation ratio is 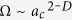. -Furthermore, the surface area is obtained by integrating the adsorption isotherm from *N*_*V*_*(S)* to the maximum value 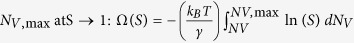.

When one plots ln(Ω) vs. ln(*a*_*c*_), a straight line is seen at some range of *a*_*c*_, typically between about 1–20 nm. The slope of the straight line is then equal to 2-*D*. In order for this method to work properly, the adsorption isotherm has to be measured up to sufficiently high saturation ratios.

### Fractal FHH theory

Avnir and Jaroniec^12^ derived, based on the Dubinin–Radushkeviech adsorption equation, an isotherm that is formally similar to the FHH isotherm: *N*_*V*_ ~ (ln *S*)^*D−3*^. It was later shown^25^ that this result is obtained using the FHH approach with *B* = 3, and incorporating the effect of capillary condensation in surface pores with help of the Kelvin equation. As *B* = 3 implies a pure van der Waals interaction between the adsorbent and the adsorbate, the fractal FHH approach is normally used to determine the fractal dimension from a nitrogen isotherm. It is best done by plotting the data as ln(−ln(*S*)) vs. ln *N*_*V*_ (so-called FHH plot) and fitting a straight line to a portion of the measurement data. To get a proper result, the adsorbed layer needs to be thicker than a monolayer, in a regime where the pores are being filled by the adsorbate^26^,^27^,^28^. Complete filling of the pores can often be seen as a change of slope in the FHH plot. For example, in most of the data sets shown in Supplementary Figure 3, the slope to the left of the selected data points is gentler than in the capillary condensation regime, indicating that all pores have been filled (whereas in the sub-monolayer adsorption regime to the right of the selected data points, the slope tends to be steeper).

### Experimental adsorption data

Water^6^,^16^,^29^,^30^,^31^ and nitrogen^6^,^16^,^31^,^32^,^33^ adsorption data sets used for fitting the FHH and fractal FHH isotherms and performing the thermodynamic fractal analysis were obtained from the literature. Monolayer volumes were taken from the experimental references when available and otherwise determined based on the reported adsorbent surface area, and literature value^34^ for the adsorbate cross-sectional area. All fits to the experimental data sets are shown in SI.

### Uncertainty analyses

The uncertainties of *B*_CCN_ shown in Table 1 were obtained from Kumar *et al.*^4^, except for the volcanic dusts, where we assumed 4% uncertainty based on the maximum uncertainty of 3.6% in the experiments of Kumar *et al.*^4^. The uncertainty of *B*_CCN_ originates from possible error sources in the measurements of the critical supersaturation and the dry size of the mineral particles such as non-spherical shapes^4^. It is worthwhile to note here that the dry sizes were determined using a differential mobility analyser which is sensitive to the macrostructure of the particle but not the microstructure, so the effect of particle porosity on the experimental dry diameter is negligible.

The uncertainties of *B*/(3*-D*), *D*_N2_(TD), and *D*_N2_(f-FHH) in Table 1 are based on the uncertainty estimates of the linear regressions shown in the SI. The uncertainties of the *D* values in Table 1 were obtained by combining the uncertainties of *B*_CCN_ and *B*/(3*-D*).

The error bars for S* (experimental) in Fig. 1 represent the uncertainty in the supersaturation of the CCN instrument, with the exception of MSH volcanic dust, where the uncertainty is determined by the two *B*_CCN_ values shown in Table 1. The error bars for the uncorrected S* (adsorption) were calculated from the uncertainties of *B*/(3*-D*) in Table 1 (they are smaller than the symbols in Fig. 1). The error bars for the fractal corrected S* (adsorption) were determined using the individual *D*_N2_(TD) and *D*_N2_(f-FHH) values given in Table 1.

## Additional Information

**How to cite this article**: Laaksonen, A. *et al.* Surface fractal dimension, water adsorption efficiency, and cloud nucleation activity of insoluble aerosol. *Sci. Rep.* **6**, 25504; doi: 10.1038/srep25504 (2016).

## Supplementary Material

Supplementary Information

## Figures and Tables

**Table 1:** Determination of the fractal dimension using three different methods.

	*B*_CCN_	*B/(3-D)*	*D*	*D*_N2_(TD)	*D*_N2_(f-FHH)
Na-Montmorill.	1.08 ± 0.03	2.38 ± 0.17	2.55 ± 0.20	2.60 ± 0.04	2.51 ± 0.06
Illite	1.12 ± 0.04	2.52 ± 0.11	2.55 ± 0.14	2.69 ± 0.04	2.39 ± 0.05
Calcite	1.30 ± 0.03	2.29 ± 0.06	2.43 ± 0.08	2.46 ± 0.03	2.48 ± 0.02
Quartz	1.36 ± 0.03	2.97 ± 0.05	2.54 ± 0.07	2.63 ± 0.03	2.66 ± 0.04
Volcanic (EC)	1.27 ± 0.05	1.89 ± 0.03	2.35 ± 0.10	2.49 ± 0.11	2.27 ± 0.04
Volcanic (MSH)	1.29–1.36 ± 0.05	2.00 ± 0.04	2.34–2.37 ± 0.11	2.43 ± 0.03	2.47 ± 0.05

The second column shows the values of the parameter *B* determined by Kumar *et al.*^4^ and Lathem *et al.*^15^ based on CCN activation measurements. The third column gives *B*/(3-*D*) as determined from water adsorption isotherms, and the fourth column presents the surface fractal dimensions calculated from the two preceding columns. The fifth and the sixth columns give the *D*-values determined using the thermodynamic and the fractal FHH approaches, respectively.

## References

[b1] Hoose C. & Möhler O. Heterogeneous ice nucleation on atmospheric aerosols: a review of results from laboratory experiments. Atmos. Chem. Phys. 12, 9817–9854, 10.5194/acp-12-9817-2012 (2012).

[b2] Sorjamaa R. & Laaksonen A. The effect of H_2_O adsorption on cloud drop activation of insoluble particles: A theoretical framework. Atmos. Chem. Phys. 7, 6175–6180, 10.5194/acp-7-6175-2007 (2007).

[b3] Kumar P., Sokolik I. N. & Nenes A. Parametrization of cloud droplet formation for global and regional models: Including adsorption activation from insoluble CCN. Atmos. Chem. Phys. 9, 2517–2532, 10.5194/acp-9-2517-2009 (2009).

[b4] Kumar P., Sokolik I. N. & Nenes A. Measurements of cloud condensation nuclei activity and droplet activation kinetics of fresh unprocessed regional dust samples and minerals. Atmos. Chem. Phys. 11, 3527–3541, 10.5194/acp-11-3527-2011 (2011).

[b5] Hatch C. D., Greenaway A. L., Christie M. J. & Baltrusaitis J. Water adsorption constrained Frenkel–Halsey–Hill adsorption activation theory: Montmorillonite and illite. Atmos. Environ. 87, 26–33, 10.1016/j.atmosenv.2013.12.040 (2014).

[b6] Hung H.-M., Wang K.-C. & Chen J.-P. Adsorption of nitrogen and water vapor by insoluble particles and the implication on cloud condensation nuclei activity. J. Aerosol Sci. 86, 24–31, 10.1016/j.jaerosci.2015.04.002 (2015).

[b7] Frenkel J. Kinetic theory of liquids, pp. 332–339 (Oxford University Press, 1946).

[b8] Halsey G. Physical adsorption on non-uniform surfaces. J. Chem. Phys. 16, 931–937, 10.1063/1.1746689 (1948).

[b9] Hill T. L. Physical adsorption and the free volume model for liquids. J. Chem. Phys. 17, 590, 10.1063/1.1747341 (1949).

[b10] Mandelbrot B. The fractal geometry of nature (Macmillan, 1983).

[b11] Sokołowska Z. & Sokołowski S. Fractal approach to adsorption/desorption processes on environmental surfaces in Biophysical chemistry of fractal structures and processes in environmental systems (eds Senesi N. & Wilkinson K. J. ) 179–220 (IUPAC, 2008).

[b12] Avnir D. & Jaroniec M. An isotherm equation for adsorption on fractal surfaces of heterogeneous porous materials. Langmuir 5, 1431–1433, 10.1021/la00090a032 (1989).

[b13] Szöri M., Tobias D. J. & Roselová M. Microscopic wetting of mixed self-assembled monolayers: a molecular dynamics study. J. Phys. Chem. B 113, 4161–4169, 10.1021/jp8074139 (2009). 19243138

[b14] Laaksonen A. A unifying model for adsorption and nucleation of vapors on solid surfaces. J. Phys. Chem. A 119, 3736–3745, 10.1021/acs.jpca.5b00325 (2015). 25831213

[b15] Lathem T. L., Kumar P., Nenes A., Dufek J., Sokolik I. N., Trail M. & Russell A. Hygroscopic properties of volcanic ash. Geophys. Res. Lett. 38, L11802, 10.1029/2011GL047298 (2011).

[b16] Malandrini H., Sarraf R., Faucompré B., Partyka S. & Douillard J. M. Characterization of quartz particle surfaces by immersion calorimetry. Langmuir 13, 1337–1341, 10.1021/la951014z (1997).

[b17] Ramachandran G. & Reist P. C. Characterization of morphological changes in agglomerates subject to condensation and evaporation using multiple fractal dimensions. Aerosol Sci. Tech. 23, 431–442, 10.1080/02786829508965326 (1995).

[b18] Kanji Z. A., Florea O. & Abbatt J. P. D. Ice formation via deposition nucleation on mineral dust and organics: dependence of onset relative humidity on total particulate surface area. Environ. Res. Lett. 3, 025004, 10.1088/1748-9326/3/2/025004 (2008).

[b19] Knopf D. A. & Alpert P. A. A water activity based model of heterogeneous ice nucleation kinetics for freezing of water and aqueous solution droplets. Farad. Discuss. 165, 513–534, 10.1039/c3fd00035d (2013). 24601020

[b20] Alpert P. A. & Knopf D. A. Analysis of isothermal and cooling-rate-dependent immersion freezing by a unifying stochastic ice nucleation model. Atmos. Chem. Phys. 16, 2083–2107, 10.5194/acp-16-2083-2016 (2016).

[b21] Marcolli C. Deposition nucleation viewed as homogeneous or immersion freezing in pores and cavities. Atmos. Chem. Phys. 14, 2071–2104, 10.5194/acp-14-2071-2014 (2014).

[b22] Laaksonen A. & Malila J. An adsorption theory of heterogeneous nucleation of water vapour on nanoparticles. Atmos. Chem. Phys. 16, 135–143, 10.5194/acp-16-135-2016 (2016).

[b23] Hertzog M., Graf H.-F., Textor C. & Oberhuber M. The effect of phase changes of water on the development of volcanic plumes. J. Volcanol. Geotherm. Res. 87, 55–74, 10.1016/S0377-0273(98)00100-0 (1998).

[b24] Neimark A. V. Thermodynamic method for calculating surface fractal dimension. JETP Lett. 51, 607–610 (1990).

[b25] Pfeifer P., Cole M. & Krim J. Multilayer adsorption on a fractally rough surface. Pfeifer, Cole, and Krim reply. Phys. Rev. Lett. 65, 663, 10.1103/PhysRevLett.65.663 (1990). 10039830

[b26] Ismail I. M. K. & Pfeifer P. Fractal analysis and surface roughness of nonporous carbon fibers and carbon blacks. Langmuir 10, 1532–1538, 10.1021/la00017a035 (1994).

[b27] Sahouli B., Blacher S. & Brouers F. Applicability of the fractal FHH equation. Langmuir 13, 4391–4394, 10.1021/la962119k (1997).

[b28] Esquena J., Solans C. & Llorens J. Nitrogen sorption studies of silica particles obtained in emulsion and microemulsion media. J. Colloid Interface Sci. 235, 291–298, 10.1006/jcis.2000.6773 (2000). 11254266

[b29] Branson K. & Newman A. C. D. Water sorption on Ca-saturated clays: I. Multilayer sorption and microporosity in some illites. Clay Miner. 18, 177–287 (1983).

[b30] Morimoto T., Kishi J., Okada O. & Kadota T. Interaction of water with the surface of calcite. Bull. Chem. Soc. Jpn. 53, 1918–1921, 10.1246/bcsj.53.1918 (1980).

[b31] Delmelle P., Villiéras F. & Pelletier M. Surface area, porosity and water adsorption properties of fine volcanic ash particles. Bull. Volcanol. 67, 160–169, 10.1007/s00445-004-0370-x (2005).

[b32] Pernyeszi T. & Dékány I. Surface fractal and structural properties of layered clay minerals monitored by small-angle X-ray scattering and low-temperature nitrogen adsorption experiments. Colloid Polymer Sci. 281, 73–78, 10.1007/s00396-002-0758-0 (2002).

[b33] Tsai W.-T. Microstructural characterization of calcite-based powder materials prepared by planetary ball-milling. Materials 6, 3361–3372, 10.3390/ma6083361 (2013). PMC5521309 28811439

[b34] McClellan A. L. & Harnsberger H. F. Cross-sectional areas of molecules adsorbed on solid surfaces. J. Colloid Interface Sci. 23, 577–599, 10.1016/0021-9797(67)90204-4 (1967).

