# Supplementary material for: Surface fractal dimension, water adsorption efficiency, and cloud nucleation activity of insoluble aerosol
Source: Sci Rep. 2016 May 3;6:25504. doi: 10.1038/srep25504 (PMC4853788; doi:10.1038/srep25504)
Supplement: Supplementary Information [file srep25504-s1.pdf]

SUPPLEMENTARY INFORMATION

Surface fractal dimension, water adsorption efficiency, and cloud nucleation activity of insoluble aerosol

Ari Laaksonen[1,2], Jussi Malila[2], Athanasios Nenes[3,4,5,6], Hui-Ming Hung[7], Jen-Ping Chen[7]

[1]Finnish Meteorological Institute, 00101 Helsinki, Finland
[2]Department of Applied Physics, University of Eastern Finland, 70211 Kuopio, Finland
[3]School of Earth and Atmospheric Sciences, Georgia Institute of Technology, Atlanta, GA 30332, USA
[4]School of Chemical & Biomolecular Engineering, Georgia Institute of Technology, Atlanta, GA, 30332, USA
[5]Institute for Environmental Research & Sustainable Development, National Observatory of Athens (NOA), I. Metaxa & Vas. Pavlou, 15236 Palea Penteli, Greece
[6]Institute of Chemical Engineering Sciences, Foundation for Research and Technology Hellas, Stadiou Str., Platani, GR-26504 Patras, Greece
[7]Department of Atmospheric Sciences, National Taiwan University, Taipei 10617, Taiwan

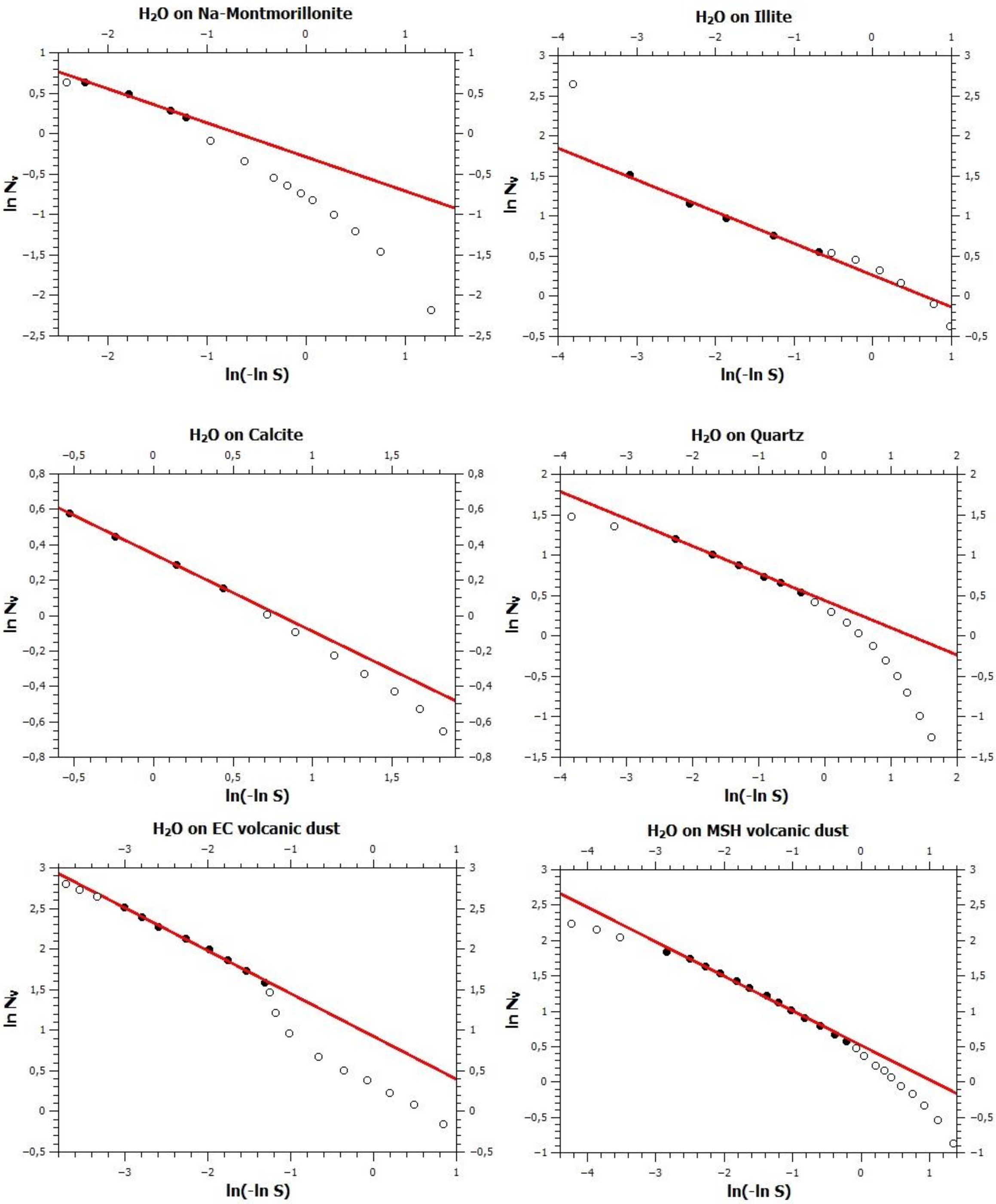

Supplementary Figure 1. FHH plots of water adsorption on Na-montmorillonite (Hung et al., 2015), Illite (Branson and Newman, 1983), Calcite (Morimoto et al, 1980), Quartz (Malandrini et al., 1997), El Chichon volcanic dust (Delmelle et al., 2005), and Mt. St. Helens volcanic dust (Delmelle et al., 2005). The *B/(3-D)*-values shown in Table 1 are obtained from the slopes of the linear fits (red lines) to the data points indicated with full circles.

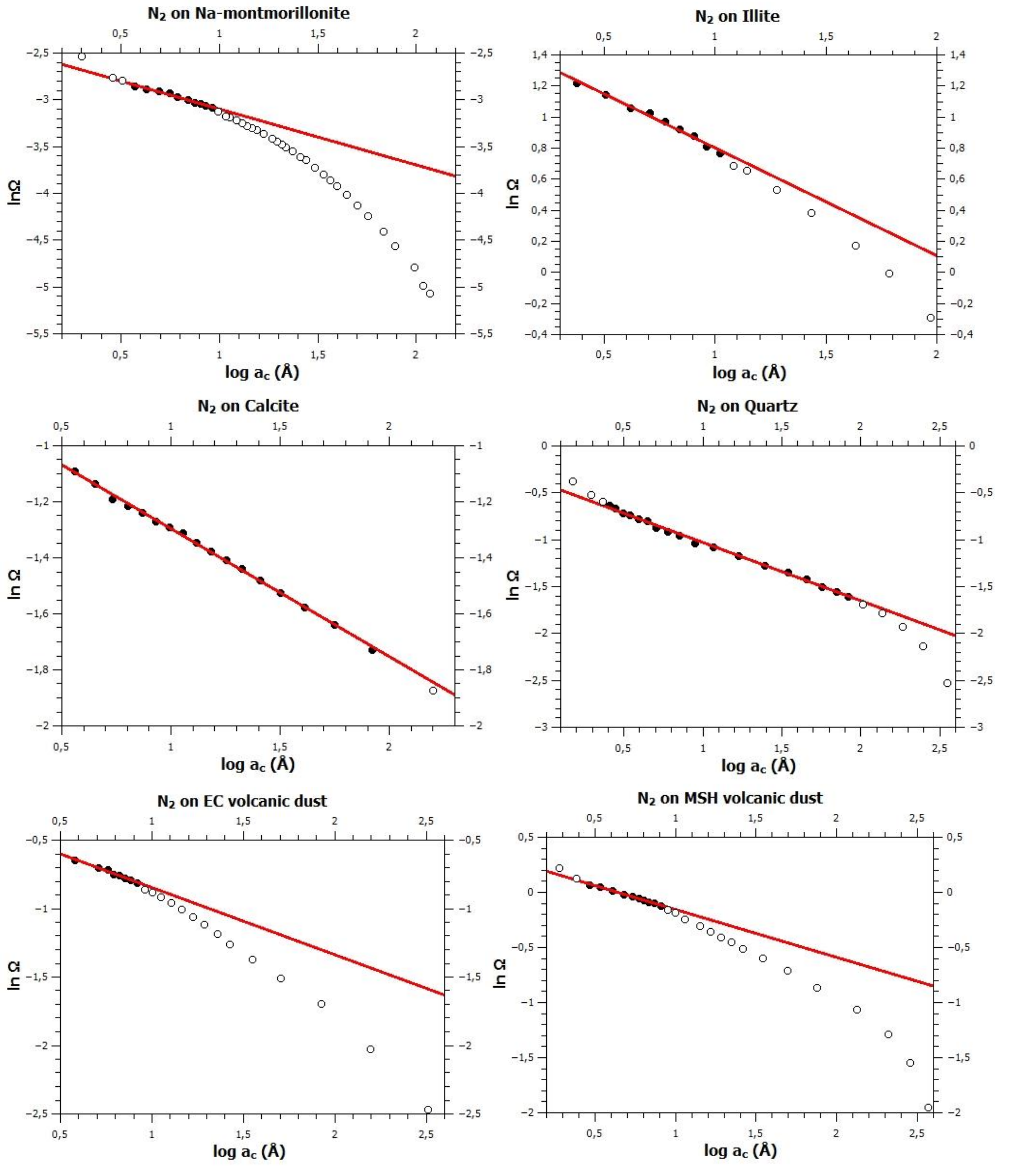

Supplementary Figure 2. Thermodynamic fractal analysis of Na-montmorillonite (Hung et al., 2015), Illite (Pernyeszi and Dékány, 2002), Calcite (Tsai, 2013), Quartz (Malandrini et al., 1997), El Chichon volcanic dust (Delmelle et al., 2005), and Mt. St. Helens volcanic dust (Delmelle et al., 2005). The D_{N2}(TD)-values shown in Table 1 are obtained from the slopes of the linear fits (red lines) to the data points indicated with full circles.

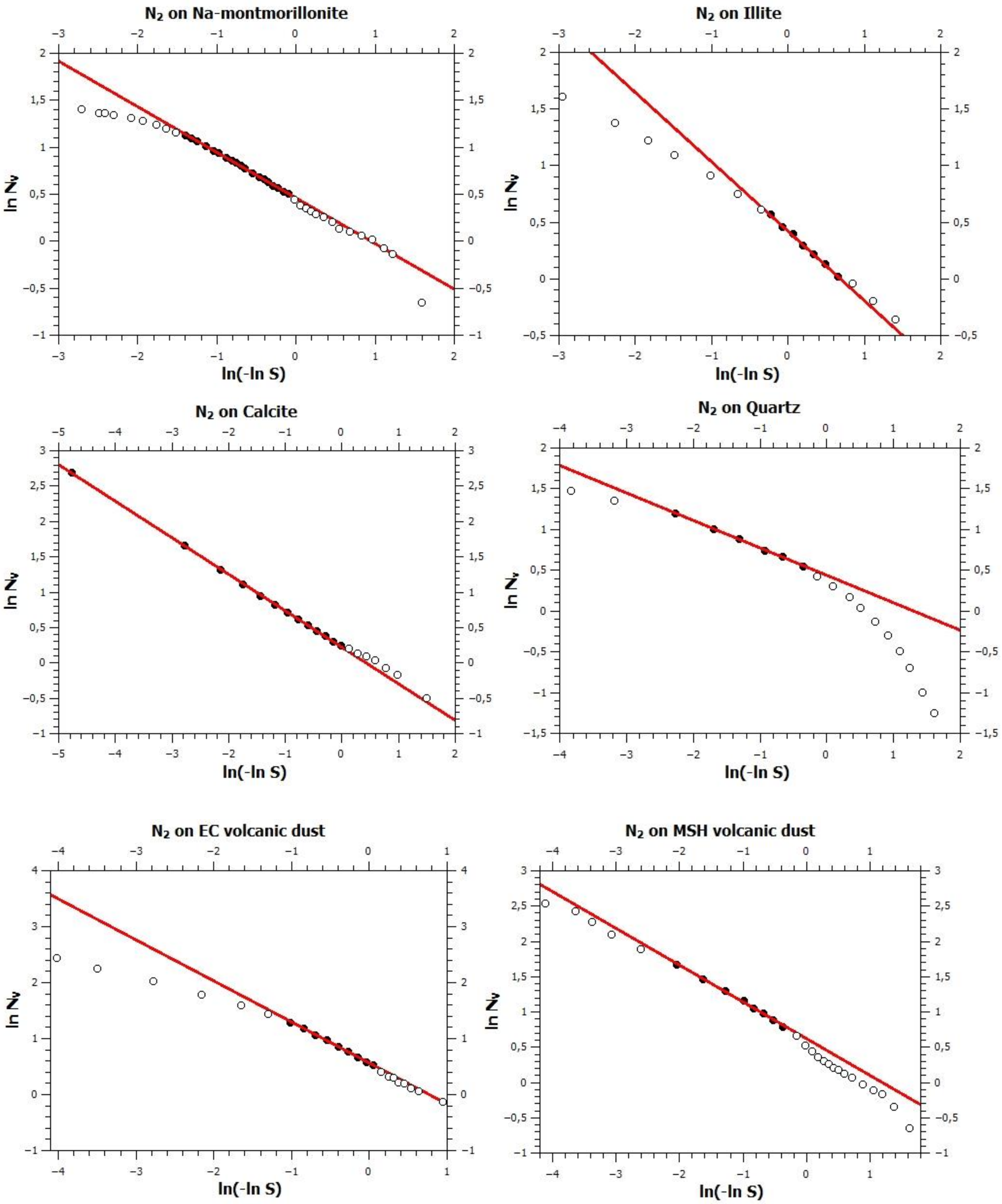

Supplementary Figure 3. FHH plots of nitrogen adsorption of Na-montmorillonite (Hung et al., 2015), Illite (Pernyeszi and Dékány, 2002), Calcite (Tsai, 2013), Quartz (Malandrini et al., 1997), El Chichon volcanic dust (Delmelle et al., 2005), and Mt. St. Helens volcanic dust (Delmelle et al., 2005). The D_{N2}(f-FHH)-values shown in Table 1 are obtained from the slopes of the linear fits (red lines) to the data points indicated with full circles.

References

Branson, K. & Newman, A.C.D. Water sorption on Ca-saturated clays: I. Multilayer sorption and microporosity in some illites. Clay Minerals 18, 177-287 (1983).

Delmelle, P., Villiéras, F. & Pelletier, M. Surface area, porosity and water adsorption properties of fine volcanic ash particles. Bull. Volcanol. 67, 160-169 (2005).

Hung, H.-M., Wang, K.-C. & Chen, J.-P. Adsorption of nitrogen and water vapor by insoluble particles and the implication on cloud condensation nuclei activity. J. Aerosol Sci. 86, 24–31 (2015).

Malandrini, H., Sarraf, R., Faucompré, B., Partyka, S. & Douillard, J.M. Characterization of quartz particle surfaces by immersion calorimetry. Langmuir 13, 1337-1341 (1997).

Morimoto, T., Kishi, J., Okada, O. & Kadota, T. Interaction of water with the surface of calcite. Bull. Chem. Soc. Jpn. 53, 1918-1921 (1980).

Pernyeszi, T. & Dékány, I. Surface fractal and structural properties of layered clay minerals monitored by small-angle X-ray scattering and low-temperature nitrogen adsorption experiments. Colloid Polymer Sci. 281, 73-78 (2002).

Tsai, W.-T. Microstructural characterization of calcite-based powder materials prepared by planetary ball-milling. Materials 6, 3361-3372 (2013).
